# MUSCLE: automated multi-objective evolutionary optimization of targeted LC-MS/MS analysis

**DOI:** 10.1093/bioinformatics/btu740

**Published:** 2014-11-11

**Authors:** James Bradbury, Grégory Genta-Jouve, J. William Allwood, Warwick B. Dunn, Royston Goodacre, Joshua D. Knowles, Shan He, Mark R. Viant

**Affiliations:** ^1^School of Computer Science and ^2^School of Biosciences, University of Birmingham, Edgbaston, Birmingham B15 2TT, UK, ^3^Manchester Institute of Biotechnology, ^4^School of Chemistry and ^5^School of Computer Science, The University of Manchester, Manchester M13 9JD, UK

## Abstract

**Summary:** Developing liquid chromatography tandem mass spectrometry (LC-MS/MS) analyses of (bio)chemicals is both time consuming and challenging, largely because of the large number of LC and MS instrument parameters that need to be optimized. This bottleneck significantly impedes our ability to establish new (bio)analytical methods in fields such as pharmacology, metabolomics and pesticide research. We report the development of a multi-platform, user-friendly software tool MUSCLE (multi-platform unbiased optimization of spectrometry via closed-loop experimentation) for the robust and fully automated multi-objective optimization of targeted LC-MS/MS analysis. MUSCLE shortened the analysis times and increased the analytical sensitivities of targeted metabolite analysis, which was demonstrated on two different manufacturer’s LC-MS/MS instruments.

**Availability and implementation:** Available at http://www.muscleproject.org.

**Contact:** info@muscleproject.org

**Supplementary information:** Supplementary data are available at *Bioinformatics* online.

## 1 Introduction

Liquid chromatography mass spectrometry (LC-MS) is widely used in analytical laboratories for measuring a range of (bio)chemicals and as the principal technology for metabolomics and proteomics. Developing new LC-MS methods and transferring existing methods between instruments and laboratories are time consuming and challenging, mostly because of the large number of LC and MS parameters that require optimization. Varying all these parameters systematically to optimize the analysis of selected chemicals is generally regarded as impossible because of the large search space.

Previously, a fully automated closed-loop strategy was reported that successfully optimized gas chromatography (GC)-MS and LC-MS methods for non-targeted metabolite analyses, resulting in increased analytical sensitivity (O’Hagan *et* *al*., 2005, 2007; Zelena *et* *al*., 2009). Although highlighting the value of closed-loop optimization in mass spectrometry, each of these implementations was for a specific manufacturer’s analytical platform. Extending this to further instruments would have required extensive reprogramming, therefore significantly limiting the deployability of this approach.

Here, we present MUSCLE (multi-platform unbiased optimization of spectrometry via closed-loop experimentation), a software tool for robust and fully automated optimization of targeted LC-MS/MS analyses. MUSCLE is instrument-manufacturer independent and requires no knowledge of computer programming to operate. Using a process called visual scripting, users create a set of configuration scripts, which instruct MUSCLE how to operate an LC-MS/MS. These scripts can be imported and exported from MUSCLE to facilitate sharing and re-use across laboratories. We demonstrate MUSCLE by optimizing the analyses of six steroids on two different manufacturer’s LC-MS/MS instruments.

## 2 Methods and implementation

MUSCLE is a stand-alone desktop application and has been tested on Windows XP, 7 and 8; see system diagram in Supplementary Figure S1. User-defined visual scripts imitate the keyboard and mouse commands that an analyst would use to manually change parameters and launch an LC-MS/MS analysis, enabling MUSCLE to control multiple LC and MS parameters on any instrument (Section 2.1). An experiment configuration contains all the information MUSCLE requires to run an automated optimization, including the user-defined LC and MS parameters to optimize and the (bio)chemicals to be analysed (Section 2.2). A multi-objective genetic algorithm (GA) optimizes the values of the LC and MS parameters, based upon the fitness of user-defined objective functions that measure, e.g. analytical sensitivity and analysis time (Section 2.3). On completion, MUSCLE presents a set of best solutions that the analyst can inspect and then select their preferred solution, a set of LC/MS parameters achieving both fast and sensitive analysis.

### 2.1 Visual scripting

Visual scripting enables direct visual references to be made to objects displayed on the screen, e.g. a ‘File’ menu item and allows MUSCLE to mimic the keyboard and mouse actions that a user would make. Here we use the Sikuli Java library (Yeh *et* *al*., 2009), providing a powerful and flexible API to allow users to create visual scripts that can: click/double click on selected objects on the screen, enter text into text fields and press selected keyboard keys, e.g. Enter. These visual scripts can be saved and later reused or modified for reuse on different analytical instruments and can be shared between laboratories using an import and export function.

### 2.2 Experiment configuration

Once visual scripts are set up to control a particular instrument, an experiment configuration can be created, which contains all the information MUSCLE requires for an automated optimization study. This includes: (i) details of the target list of (bio)chemicals to be analysed, including *m/z* values of the parent and fragment ions (e.g. Supplementary Fig. S2 and Table S1), (ii) settings for the GA including the user-defined objective functions (see Section 2.3 and Supplementary Table S2) and (iii) user-defined list of LC and MS parameters to be optimized, where each parameter has an associated visual script and minimum, maximum and step size values (e.g. Supplementary Table S3). Further details of the experiment configuration are provided in Supplementary Material.

### 2.3 Closed-loop evolutionary optimization

Closed-loop evolutionary optimization is a probabilistic search heuristic, whereby potential solutions are evaluated by conducting physical experiments (Knowles, 2009), which in the case of MUSCLE corresponds to LC-MS/MS analyses. Each solution represents a set of control parameters for the LC-MS instrument and is generated using a GA. Typically, GAs evaluate tens of thousands of solutions *in silico* during an optimization process. Because of the time constraints on evaluating each solution in an LC-MS/MS study, closed-loop optimization requires the GA to perform well when limited to just a few tens or hundreds of evaluations. To evaluate each solution, a fitness value is calculated for each of the objectives, where each objective measures the quality of the LC-MS/MS spectra obtained using the selected instrument settings. For targeted LC-MS/MS analysis, the user-selected objectives include (i) minimizing the analysis time (measured as the retention time of the last eluting target analyte, not the total analysis time); (ii) maximizing the number of analytes detected from the target list and (iii) maximizing the total peak area of these analytes. Fitness values are calculated based on the results of a custom peak detection algorithm (see Supplementary Material), which processes mzML files (Supplementary Fig. S1). This enables MUSCLE to analyse results from any LC-MS/MS instrument following conversion of the vendor specific data format to mzML. Because the three objectives are in conflict, a multi-objective GA must be used, which can efficiently find a set of Pareto optimal solutions. Typically, a large number of optimization experiments are required to achieve a highly optimized search method, which due to cost implications of conducting LC-MS/MS analyses was not feasible. We therefore opted to use the PESA-II multi-objective GA (Corne *et al*., 2001) as it is widely used, and we are familiar with configuring this algorithm for the optimization of mass spectrometry analyses. The Java library implementation of the algorithm, jMetal (Durillo *et al*., 2010), was used in this case. The values of each LC and MS parameter in the first *n* runs (where *n* is user defined) are chosen randomly. For each subsequent run, the GA decides the LC and MS parameters based upon the evaluation of previous LC-MS/MS analyses, favouring parameters that produced high fitness values. The GA maintains a set of the best solutions in an archive set and from these solutions decides on the next set of parameters by applying selection, crossover and mutation operators. If suboptimal parameters are selected by the GA, a low-quality chromatogram will result with low fitness values, which will not be added to the archive set.

The user selects the maximum number of runs to be performed, which fixes the overall time and cost of the optimization. Because of the limited number of runs, the optimization algorithm will never realistically reach the global optima but instead has a high likelihood of converging towards a local optima. The user is shown real-time results of the optimization and if the convergence towards an optimum set of parameters seems to be complete, they have the ability to pause or completely stop the optimization.

## 3 Results and discussion

We have demonstrated MUSCLE in two common laboratory scenarios, using two manufacturers’ LC-MS/MS instruments and associated software, to optimize the targeted analysis of a mixture of six steroids (Supplementary Fig. S2). First, we used MUSCLE to improve an LC-MS/MS analysis that had previously been optimized manually by an experienced analytical chemist, using a Thermo Scientific UHPLC Ultimate 3000 TSQ Vantage running under Xcalibur software V2.0.7. Second, we transferred this manually optimized method from the Thermo Scientific instrument to a Waters ACQUITY UPLC Xevo TQ LC-MS/MS running under MassLynx software V4.1 and used MUSCLE to re-optimize the LC and MS parameters.

**Fig. 1.**
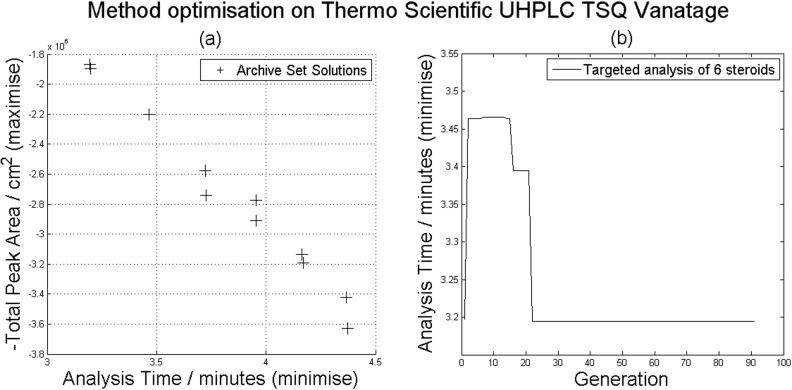
(**a**) Pareto front. The total peak area axis has been reversed for readability, each cross represents a solution with the corresponding objective values. (**b**) Generation-by-generation lowest run time in the archive set (considering the set of best solutions for which six out of six peaks are detected). The first generation was 20 randomized runs and each subsequent generation consisted of two runs

In the first study, the user selected minimum and maximum values and step sizes for each of 10 LC and MS parameters to be optimized (Supplementary Table S3 and Fig. S3) along with settings for the GA (Supplementary Table S2). Following an ∼48-h optimization, comprising 200 LC-MS/MS analyses, this fully automated approach discovered an improved set of parameters that provided a faster (34.5%) and more sensitive (10.0%) analysis than achieved manually (Supplementary Table S4). Figure 1a shows the final Pareto front after 200 analyses. Figure 1b shows how the LC analysis time decreases through the optimization process, considering the set of best solutions for which six out of six steroids are detected, plateauing around generation 20.

Following transfer of the manually optimized method from the Thermo Scientific to Waters instrument, the minimum and maximum values and step sizes for nine LC and MS parameters were selected (Supplementary Table S5) and then optimized during an ∼48-h fully automated LC-MS/MS study. Again, MUSCLE was able to discover an improved set of parameters that provided a faster (18.5%) and much more sensitive (104%) analysis (Supplementary Table S6). Supplementary Figure S4a shows the final Pareto front after 200 analyses. Supplementary Figure S4b shows how the total peak area increases through the optimization.

One limitation of MUSCLE is that an optimization is prone to finding local rather than global optimum solutions. Also the convergence of some optimizations may plateau before the maximum number of runs has been reached. To combat this, the user has the ability to view the results of the optimization generation by generation, and if they feel that MUSCLE is no longer improving the quality of the analysis, the optimization can be paused or stopped completely. A further limitation is the peak detection procedure, which is based on a relatively simple algorithm that is designed to work for data derived from a range of mass spectrometers, as described in Section 1.1.3 (Supplementary Material). However, MUSCLE has been programmed to enable the user to add alternative peak detection algorithms from a drop-down box, should the current implementation not work well for a particular dataset.

In conclusion, MUSCLE shortened the analysis times and increased the analytical sensitivities of the targeted analysis of multiple steroids on two manufacturer’s LC-MS/MS instruments in a fully automated manner and is anticipated to benefit several fields including pharmacology, metabolomics and proteomics.

## Supplementary Material

Supplementary Data
