# Supplementary material for: MUSCLE: automated multi-objective evolutionary optimization of targeted LC-MS/MS analysis
Source: Bioinformatics. 2014 Nov 11;31(6):975–7. doi: 10.1093/bioinformatics/btu740 (PMC4380028; doi:10.1093/bioinformatics/btu740)
Supplement: Supplementary Data [file supp_31_6_975__index.html]

MUSCLE: Automated Multi-objective Evolutionary Optimisation of Targeted LC-MS/MS Analysis — MUSCLE: automated multi-objective evolutionary optimization of targeted LC-MS/MS analysis — MUSCLE: automated multi-objective evolutionary optimization of targeted LC-MS/MS analysis — Supplementary Data 

# MUSCLE: automated multi-objective evolutionary optimization of targeted LC-MS/MS analysis

## Supplementary Data

files

**Files in this Data Supplement:**

- Supplementary Data - pdf file
