# Supplementary material for: MUSCLE: automated multi-objective evolutionary optimization of targeted LC-MS/MS analysis
Source: Bioinformatics. 2014 Nov 11;31(6):975–7. doi: 10.1093/bioinformatics/btu740 (PMC4380028; doi:10.1093/bioinformatics/btu740)
Supplement: Supplementary Data [file supp_btu740_SI.pdf]

Supplementary Data to MUSCLE: Automated Multi-objective Evolutionary Optimisation of Targeted LC-MS/MS Analysis

James Bradbury [1], Grégory Genta-Jouve [2], J. William Allwood [2], Warwick B. Dunn [2], Royston Goodacre [3], Joshua D. Knowles [4], Shan He [1], and Mark R. Viant [2]

[1] School of Computer Science, University of Birmingham, UK

[2] School of Biosciences, University of Birmingham, UK

[3]Manchester Institute of Biotechnology and School of Chemistry, University of Manchester, UK

[4] School of Computer Science, University of Manchester, UK

Availability: Available at http://www.muscleproject.org
Contact: info@muscleproject.org

Contents

1 Methods **2**
- 1.1 MUSCLE Software 2
  - 1.1.1 Closed-loop optimisation process 2
  - 1.1.2 Genetic algorithm representation 3
  - 1.1.3 Peak detection algorithm 3
- 1.2 Biochemicals on target list 4
- 1.3 Liquid chromatography (LC) parameters 5

2 Results **5**
- 2.1 LC-MS/MS method optimisation on Thermo Scientific UHPLC TSQ Vantage 6
- 2.2 LC-MS/MS method transfer and optimisation on Waters ACQUITY UPLC Xevo TQ 8

1 Methods

1.1 MUSCLE Software

Figure S1 shows the overall system diagram of MUSCLE. A user creates an Experiment Configuration which contains all of the information required by the algorithm to perform an optimisation study. This includes:

1. A set of parameters that are to be optimised along with minimum and maximum values and a step size for each, as well as a visual script to be used to enter values for these parameters.

2. Some general optimisation settings; the total number of analyses to evaluate and the number of analyses to be randomly selected at the start of the optimisations. Also some settings for the genetic algorithm (GA) need to be captured, these are population size, and mutation and crossover rates. The GA settings are given a default value to help the user.

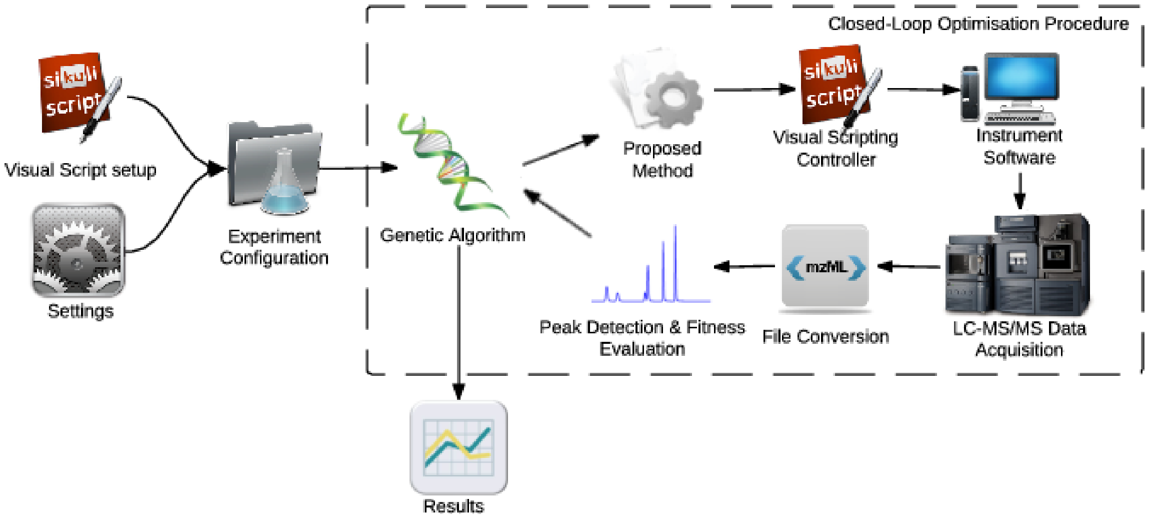

Figure S1: MUSCLE system diagram

1.1.1 Closed-loop optimisation process

The Experiment Configuration contains all of the information required to conduct an optimisation study. Once it is complete, it is used by MUSCLE to perform the fully automated closed-loop optimisation. The procedure for the closed-loop optimisation is as follows:

1. The GA decides on a value for each of the user selected LC and MS parameters defined in the Experiment Configuration for the next run[1].

[1] The values for the first n runs are chosen randomly, where n is defined by the user in the Experiment Configuration

2. The set of values for these instrument control parameters forms a proposed method.
3. This proposed method is then passed to the Visual Scripting Controller, which obtains all the visual scripting commands from the Experiment Configuration.
4. The Visual Scripting controller then executes the visual scripting commands, imitating mouse and keyboard actions to change the control parameters on the appropriate instrument software.
5. Once the full set of control parameter values are entered, another user defined visual script is used to initiate the LC-MS/MS data acquisition.
6. Once the data acquisition has finished, the native data output file from the LC-MS/MS is converted to an .mzML file.
7. This .mzML file is passed to MUSCLE's peak detection algorithm (see section 1.1.3) which outputs a list of detected peaks. This list of peaks is then used to calculate fitness values for each of the objectives; minimising the analysis time (measured as the retention time of the last eluting target metabolite), maximising the number of analytes detected from the target list and maximising the total peak area of these analytes.
8. The fitness information is passed back to the GA, and if the data output for the proposed method is considered to be better for at least one objective than that is maintained in an *archive* set (or set of best solutions), it is added to the archive.
9. The GA then uses the solutions in the archive and a series of operators (crossover, selection and mutation) to generate a new LC-MS/MS method to evaluate.
10. The whole process is repeated until the maximum number of analyses (as defined in the Experiment Configuration) is reached.

1.1.2 Genetic algorithm representation

The solutions are encoded for the GA using a binary representation. This means that a solution is represented by a single binary string containing a smaller substring for each parameter. To get a control parameter value the relevant binary substring is converted to a decimal number.

Representing the solution using a binary string means that genetic operators can be easily applied. The crossover operator mimics breeding and takes two solutions (parent 1 and 2 which are represented as binary strings) of length y and picks a random point x such that $x < y$. The two binary strings are then cut at that point and a child solution is generated by taking the digits from before x from parent 1 and combining it with the digits after point x from parent 2, thus creating a new solution that is a combination of its two parent solutions. The mutation operator mimics genetic mutation by choosing a random binary digit and *flipping* it, so if the digit is a 1, it is flipped to become a 0 and vice-versa.

1.1.3 Peak detection algorithm

MUSCLE uses a custom written algorithm to detect peaks from an mzML file. The procedure (for each scan) is as follows:

1. Smooth the signal to reduce the potential that noise detected as small peaks. The smoothing is done using the unweighted sliding-average smoothing algorithm, which replaces each point in the signal with the average of m adjacent points, where m is a positive integer called the *smooth width* and can be chosen by the user (the default value is set as 50). This smoothing procedure is repeated 3 times.

2. Check the first part of the signal to calculate the size of the background noise, the value is stored as an amplitude threshold.

3. Calculate the first derivative of the signal.

4. Find the points at which there are zero crossings in the first derivative. This indicates the slope changes direction and therefore that a peak is present.

5. Calculate the maximum slope of the peak.

6. If the slope of the peak is sufficiently high i.e. above a pre-defined threshold, and if the height of the peak is greater than the pre-calculated noise amplitude threshold, integrate the peak.

7. Store the height, width, chromatographic retention time and area of the peak and add to a global peak list.

N.B. if more than 1 peak is detected per scan, only the highest peak is added to the peak list.

1.2 Biochemicals on target list

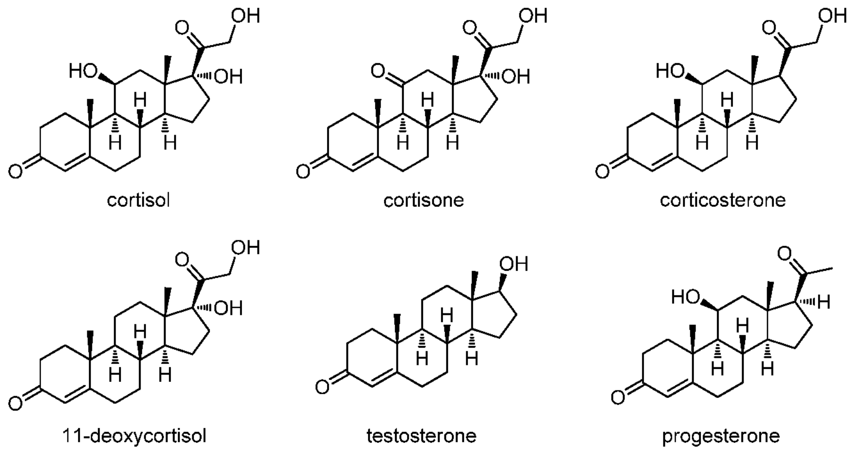

Figure S2: Selected steroids for the LC-MS/MS optimisation

Figure S2 presents the structures of the biochemicals on the target list for LC-MS/MS analysis. Although these compounds belong to the same family, they don't have the same affinity

for the the reverse phase chromatographic column. While cortisol and cortisone are relatively polar, progesterone and testosterone are fairly apolar. Corticosterone and 11-deoxycortisol are two diastereoisomers often co-eluting in standard chromatographic conditions, hence these two compounds have been chosen to demonstrate the ability of MUSCLE to separate even structurally similar compounds.

For any LC-MS/MS analysis, a list of mass transitions must be pre-defined by the analyst (Table S1). A mass transition describes the m/z values of the parent ion and fragment ion, for each biochemical on the target list. The intensity of the fragment ion signal is used to quantify the biochemical.

Table S1: List of mass transitions, parent ion → fragment (quantifier) ion used to identify and quantify each of the six steroids

Steroid	Mass transition (m/z)	Collision energy
Cortisone	361.3 → 163.1	20%
Cortisol	363.2 → 121.1	21%
Corticosterone	347.3 → 329	10%
11-deoxycortisol	347.3 → 97.1	24%
Testosterone	289.2 → 97.1	22%
Progesterone	315.2 → 97.1	18%

1.3 Liquid chromatography (LC) parameters

During the optimisation, the GA is allowed to change several user-defined LC and MS parameters. A generic chromatogram gradient is shown in Figure S3. The first parameter (1) corresponds to the time of the starting conditions. The holding duration (2) indicates how long the starting condition will remain unchanged. The third parameter (3) indicates when the gradient starts. The duration of the gradient is controlled by parameter 4 (ramping duration) and when the gradient reaches the final conditions (5), the conditions are unchanged for a duration defined by 6. The user is able to set constraints on the minimum and maximum values of these parameters. For the studies presented in this paper, the duration defined by parameter 6 was constrained so that is was at least 2 minutes. This is so that the column can be sufficiently rinsed before the next analysis.

2 Results

Table S2 shows the GA settings used for both optimisation studies. The total number of analyses was 200, with the first 20 having randomly selected values for the LC and MS parameters (within the ranges defined by the user). The population size represents the number of solutions that are evaluated in each iteration of the genetic algorithm before the archive set is refreshed. In other words, after every 2 LC-MS/MS analyses, the 2 solutions are considered for the archive set (maintained set of best solutions) and are added to it if they represent a better solution (in terms

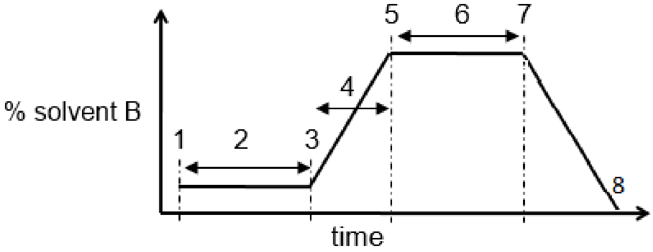

Figure S3: Generic LC gradient showing how the percentage of solvent B (in a two solvent analysis) changes over time. For the studies presented, parameters 3, 4, 5 & 6 are chosen by the genetic algorithm during the optimisation. The values for parameters 1, 2, 7 & 8 are unchanged throughout the optimisation, to ensure that no peaks are eluted at the very start of a run, and that the column is sufficiently washed and that no cross-contamination occurs between runs. Once the analyst has chosen their preferred optimal method, these parameters can be changed accordingly. **Key:** (1) start, (2) initial hold duration, (3) gradient ramp start, (4) gradient ramping duration, (5) gradient final hold start, (6) gradient final holding duration, (7) gradient final hold end, (8) end.

of the fitness values for the objectives being optimised). The crossover and mutation rates are the probabilities that the genetic algorithm applies to the crossover/mutation operator (see section 1.1.2).

Table S2: GA settings for both optimisation studies.

Number of Analyses	200
Number of Initial Random Methods (n)	20
Population Size	2
Crossover Rate	0.7
Mutation Rate	0.2

2.1 LC-MS/MS method optimisation on Thermo Scientific UHPLC TSQ Vantage

Table S3 shows each of the LC and MS instrument parameters that were selected for optimisation on the Thermo Scientific UHPLC coupled to a TSQ Vantage LC/MS-MS. Each parameter is named according to the instrument software. For each parameter, a minimum, a maximum and a step size value are selected, which defines the possible values that can be entered for each parameter. Taking Spray Voltage as an example, the minimum value is 3000, the maximum value is 4500 and the step size is 250, this gives 7 possible values: 3000, 3250, 3500, 3750, 4000, 4250, 4500.

The total search space (the total number of unique combinations of each of the parameters) given the values in Table S3 is 2.89×10^9. Assuming a total LC-MS/MS analysis time of ca. 5 min, searching all of this space would require ca. 27,500 years.

Table S3: Thermo Scientific UHPLC TSQ Vantage LC-MS/MS optimisation parameters.

Parameter	Minimum	Maximum	Step Size	Units
Auxillary Gas Pressure	10	40	5	a.u.
Capillary Temperature	280	350	10	°C
Ion Sweep Gas Pressure	0	15	3	a.u.
Sheath Gas Pressure	0	15	3	a.u.
Spray Voltage	3000	4500	250	mV
Vaporizer Temperature	150	280	10	°C
Gradient Ramp Start	1	6	0.5	min
Gradient Ramping Duration	1	6	0.5	min
Gradient Final Hold Start	1	6	0.5	min
Gradient Final Holding Duration	1	6	0.5	min

Table S4 shows the results of the method optimisation experiment on a Thermo Scientific UHPLC TSQ Vantage. The column labelled 'starting' shows the values for all of the control parameters for the manually optimised method (conducted by an experienced analytical chemist). The subsequent columns show the control parameter values and the objective values for: The MUSCLE optimised experiment with the best analysis time, the MUSCLE optimised experiment with the best total peak area, and preferred method chosen by the analyst. The analyst chosen run stems from an important feature of the software. MUSCLE does not produce a single optimised method, it instead produces a set of methods that are maintained in the archive set. This allows the analyst to choose which of the methods is most preferable, be it a method with a short analysis time, a high total peak area or a trade-off between the two, dependant upon their requirements.

In this case the analyst has chosen the method with the shortest analysis time which is 3.19 minutes. That represents a 34.5% decrease in analysis time compared to the manually optimised (starting) method.

Table S4: Thermo Scientific UHPLC TSQ Vantage LC-MS/MS method optimisation results.

Parameter	Starting (manually optimised)	Best Run Time (MUSCLE optimised)	Best Peak Area (MUSCLE optimised)	Preferred method selected by analyst
Auxillary Gas Pressure (a.u.)	35	40	10	40
Capillary Temperature (°C)	290	350	350	350
Ion Sweep Gas Pressure (a.u.)	3	15	0	15
Sheath Gas Pressure (a.u.)	15	15	15	15
Spray Voltage (mV)	4000	4000	3250	4000
Vaporizer Temperature (°C)	270	180	210	180
Gradient Ramp Start (min)	2.0	1.0	2.5	1.0
Gradient Ramping Duration (min)	4.0	2.0	4.5	2.0
Gradient Final Hold Start (min)	5.5	5.5	5.5	5.5
Gradient Final Holding Duration (min)	6.0	6.0	6.0	6.0
Objective Values				
Total Peak Area (counts)	1.70×10^6	1.87×10^6	3.07×10^6	1.87×10^6
Analyses Time (min)	4.87	3.19	5.18	3.19
Number of Peaks from target list	6 (of 6)	6 (of 6)	6 (of 6)	6 (of 6)

2.2 LC-MS/MS method transfer and optimisation on Waters ACQUITY UPLC Xevo TQ

MUSCLE can be used to transfer methods between instruments and re-optimise the analysis. In this optimisation, the same biological sample is used as for the optimisation in section 2.1. As the two instruments used have different control parameters and different software, the analyst must choose which LC-MS/MS parameters they wish to optimise on the second instrument as in most cases there is no formal one-to-one mapping of control parameters. A new visual script for each parameter must also be created.

An example of two parameters that have the same functionality between the two instruments are *Spray Voltage* on the Thermo Scientific TSQ Vantage and *Capillary Voltage* on the Waters Xevo TQ. To effectively transfer the method, the *Capillary Voltage* control parameter must be included in the optimisation. It is also worth noting that the two instruments use different units for these parameters. On the Thermo Scientific instrument the value is in millivolts whereas the Waters instrument uses Volts. The analysts must therefore be careful when choosing the minimum and maximum values to be used in the optimisation.

Table S5 shows each of the control parameters that were selected for optimisation on the Waters ACQUITY UPLC Xevo TQ LC-MS/MS. Each parameter is named according to the instrument software. For each parameter, a minimum, a maximum and a step size value are selected which defines the possible values that can be entered for each parameter. Taking Cone Voltage as an example, the minimum value is 3.0, the maximum value is 12.0 and the step size is 1.0, this gives 10 possible values: 3.0, 4.0, 5.0, 6.0, 7.0, 8.0, 9.0, 10.0, 11.0, 12.0.

The total search space (the total number of unique combinations of each of the parameters) given the values in Table S5 is 1.01×10^9.

Table S5: Waters ACQUITY UPLC Xevo TQ LC-MS/MS optimisation parameters

Parameter	Minimum	Maximum	Step size	Units
Capillary Voltage	3.0	5.0	0.1	V
Cone Gas Flow	0.0	5.0	1.0	$L.h^{-1}$
Cone Voltage	3.0	12.0	1.0	V
Desolvation Gas Flow	800	1600	200	$L.h^{-1}$
Desolvation Temperature	250	500	25	°C
Gradient Ramp Start	1	6	0.5	min
Gradient Ramping Duration	1	6	0.5	min
Gradient Final Hold Start	1	6	0.5	min
Gradient Final Holding Duration	1	6	0.5	min

Table S6 shows the results of the method transfer and optimisation experiment on a Waters ACQUITY UPLC Xevo TQ. The corresponding final Pareto front after 200 analyses is shown in Figure S4a. The increase in the total peak area through the optimisation study is presented in Figure S4b.

In this case the analyst has chosen the method with the highest total peak area, which is 1.24×10^9. That represents a 104% increase in total peak, and an 18.5% decrease in analysis time when compared to the initial optimised (starting) method.

Table S6: Results of the method transfer optimisation to Waters ACQUITY UPLC Xevo TQ LC-MS/MS

Parameter	Starting (manually optimised)	Best Run Time (MUSCLE optimised)	Best Peak Area (MUSCLE optimised)	Preferred method selected by analyst
Capillary Voltage (V)	4	4	3.9	3.9
Cone Gas Flow (L.h^{-1})	2	3	5	5
Cone Voltage (V)	7	6	8	8
Desolvation Gas Flow (L.h^{-1})	1000	1000	800	800
Desolvation Temperature (°C)	275	250	475	475
Gradient Ramp Start (min)	2.5	2.0	1.0	1.0
Gradient Ramping Time (min)	3.5	2.5	3.5	3.5
Gradient Final Hold Start (min)	5.5	5.5	5.5	5.5
Gradient Final Holding Time (min)	6.0	6.0	6.0	6.0
Objective Values				
Total Peak Area (counts)	6.08×10^8	6.58×10^6	1.24×10^9	1.24×10^9
Analysis Time (min)	4.06	3.27	3.31	3.31
Number of Peaks	6 (of 6)	6 (of 6)	6 (of 6)	6 (of 6)

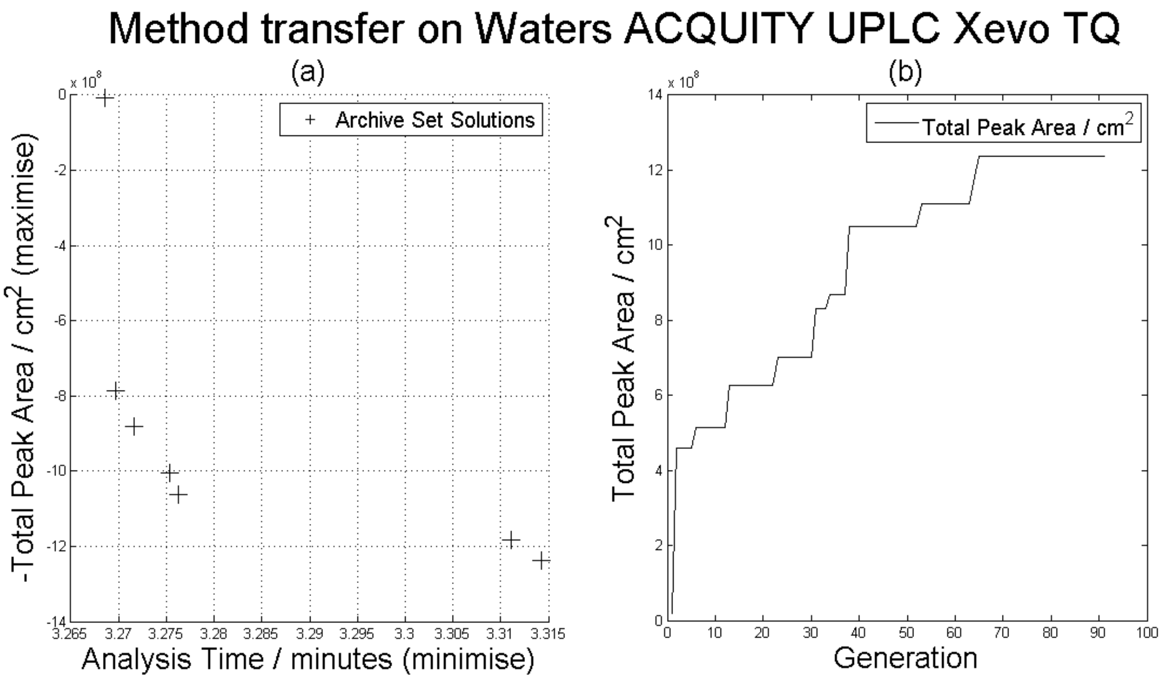

Figure S4: (a) Pareto front of the final archive set (solutions with all 6 peaks detected) for the method transfer experiment. (b) Generation-by-generation highest peak area in the archive set (solutions with 6 peaks). The first generation was 20 randomised runs, and each subsequent generation consisted of 2 runs.
